# Indirect exposure to organophosphate pesticides and its possible growth disorders in children of farmers in Northwest Mexico

**DOI:** 10.1007/s00420-026-02201-x

**Published:** 2026-01-27

**Authors:** Estefanía Ochoa-Ruiz, Aarón Israel Moreno-García, Daniel Alejandro Licea-Espinoza, Gisela Pineda-García, Krysta Paola Carranza-Ambriz, Kenya Karina Soto-Rodriguez, Arnold García-Ledezma, José Manuel Cornejo-Bravo, Ana Laura Martínez, Eleonora Mondani, Aracely Serrano-Medina

**Affiliations:** 1 Facultad de Medicina y Psicología, Universidad Autónoma de Baja California, Calzada Universidad 14418, Parque Industrial Internacional, C.P. 22424 Tijuana, B.C. Mexico; 2 Facultad de Ciencias Químicas e Ingeniería, Universidad Autónoma de Baja California, Calzada Universidad 14418, Parque Industrial Internacional, C.P. 22424 Tijuana, Mexico; 3 Centro Universitario de Ciencias de la Salud, Universidad de Guadalajara, Sierra Mojada 950, Independencia Oriente, C.P. 44340 Guadalajara, Jal. Mexico; 4 Department of Medicine and Surgery, School of Medicine and Surgery, University of Parma, A. Gramsci Street N. 14, 43126 Parma, Italy

**Keywords:** Cholinesterases, Endocrine disruptors, Children, Organophosphate pesticides, Environmental exposure, Growth disorders

## Abstract

**Purpose:**

This study investigated the correlation between growth disorders and indirect pesticide exposure in children of farmers.

**Methods:**

A cross-sectional study was conducted with 134 children of farmers occupationally exposed to organophosphate pesticides in the ejido Venustiano Carranza, an agricultural community in San Quintín, Baja California, Mexico. These children were compared with a control group of 56 unexposed children. Anthropometric measurements and biochemical analyses were performed in both groups to evaluate renal, hepatic, and nutritional profiles. Hormonal profiles were quantified using ELISA techniques. Cholinesterases are quantified using known colorimetric methods. Food security was also analyzed through a dietary diversity study, focusing on households.

**Results:**

Indirectly exposed group showed a higher prevalence of stunting (18% vs. 16.4%) than controls. Food safety was adequate in both groups (X^2^ = 1.88, *p* = 0.597). The indirectly exposure children showed significantly higher calcium levels (9.99 ± 0.63 mg/dL) and significant increases in liver enzymes AST and GGT (*p* < 0.001). ALP was 1.5 times lower than in unexposed children (*p* < 0.001), showing a strong association with exposure time (*p* = 0.009). The indirectly exposed group showed lower GH and IGF-1 levels; 2.55 and 1.28 times lower than in controls, respectively. Children with indirect exposure showed a significant reduction in AChE (7211 ± 1917 vs. 8368 ± 213 U/L, *p* < 0.001) and an even greater decrease in BChE (6426 ± 1664 vs. 8025 ± 1462 U/L, *p* < 0.001).

**Conclusion:**

This work demonstrates an association between environmental exposure to organophosphate pesticides and children's growth and provides crucial insights for future research and policymaking.

## Introduction

Humans are directly exposed to pesticides in occupational, agricultural, and household activities and indirectly via environmental media, including air, water, soil, and food. People directly and/or indirectly exposed to pesticides may contract acute toxicity effects and chronic diseases (Tudi et al. 2022). Pesticide exposure is not limited to directly exposed workers but includes nearby workers, their families, and residents near growing areas. Therefore, agricultural workers are doubly exposed, both environmentally and occupationally, since living very close to cultivation areas in this sector is common. For this reason, the exposure becomes chronic (Serrano-Medina et al. 2019). Children living in rural areas are constantly exposed to risks inherent in agricultural activities, including chemical risks; pesticides and metals are chemical/toxic substances that can occur in farming environments, and their contamination in soil, water, and air represents a concern due to the various risks to children's health (Landrigan 2014; Uwizeyimana et al. 2017). Farmers’ families are potentially exposed to pesticides indirectly by take-home contamination. Farmer’s children or children who live near farmland where pesticides are used have higher exposure than children in the general population (Carly Hyland 2017).

Many of these xenobiotics have been classified as endocrine disruptors, which are compounds that can interfere with the function of the endocrine system (Maqbool et al. 2016). They mimic hormones or block their receptors, disrupting normal gland function and affecting the entire organism. They potentially induce many chronic diseases such as diabetes, obesity, reproductive abnormalities, thyroid disorders, growth disorders, and neurological developmental dysfunction (Russ and Howard 2016; Kartini et al. 2019). Kartini et al. (2019) hypothesized that pesticide exposure could be a risk factor for developing growth disorders in children living in agricultural areas through a case–control study (Kartini et al. 2019). They found no significant differences between cases and controls regarding baseline characteristics, except for the median. Insulin Growth Factor type 1(IGF-1) level was significantly lower in cases (66.73 ng/mL) than in controls (112.57 ng/mL). High levels of pesticide exposure and low levels of Insuline-Like Growth Factor 1 (IGF-1) were significantly associated with growth retardation.

On the other hand, Nascimento et al. (2018) analyzed the enzymatic activity of a rural child population. They supported the idea that exposure to environmental xenobiotics, especially pesticides and metals, may be associated with endocrine problems in children living in rural communities (Nascimento et al. 2018). However, not enough research describes pesticides' endocrine and developmental effects due to environmental exposure in rural populations, especially in the child population.

The study of organophosphate (OP) pesticide contamination in Baja California is justified by the region's significant agricultural importance and its intensive use's health and environmental implications. In areas such as the Mexicali Valley and San Quintín, where horticultural and fruit production for the national and international markets is concentrated, OPs—including dichlorvos, acephate, naled, parathion, and malathion—have historically been the most widely used compounds due to their effectiveness in pest control and their low cost (Serrano-Medina et al. 2019). However, the repeated application of these compounds increases the likelihood of accumulation in soils, water bodies, and agricultural products, posing a significant risk to ecosystems and local communities. From a health perspective, OPs act as inhibitors of the enzyme cholinesterases (Taylor 2009), which can cause broad acute and chronic health effects in agricultural workers and directly or indirectly exposed populations, with a history of documented poisoning in different regions of Mexico (Stiftung 2025). This proposal seeks to determine the presence of growth disorders in children and their association with environmental exposure to Ops by measuring the enzymatic activity of acetylcholinesterase (AChE) and butyrylcholinesterase (BChE), endocrine parameters, clinical biochemistry, and food safety in a rural child population in northwestern Mexico. Furthermore, it seeks to prevent acute and chronic poisoning in rural populations through adequate technical guidance on pesticide use and health safety precautions, essential for informing health professionals about associated environmental and health problems. All these aspects should be investigated in order to implement programs that improve the health of agricultural workers and their families. A health monitoring system is also necessary, particularly for children presenting clinical pesticide exposure symptoms. In this context, generating local scientific evidence on the magnitude and characteristics of OP contamination is essential to support the design of public policies, strengthen integrated pest management programs, and promote sustainable agricultural practices that guarantee the protection of human health and the environment.

## Materials and methods

### Study design and volunteer selection

In the San Quintin Valley, Baja California, Northwest Mexico, we contacted a daycare center to invite the children's parents to participate in the project. We selected 134 children with indirect exposure. These children belonged to parents residing in the district of Venustiano Carranza, also known as Santa María. Their parents work in agriculture, which involves pesticide application, harvesting vegetables from areas previously treated with pesticides, and packaging vegetables. Children whose parents had direct pesticide exposure for at least six months during their work were included in the study group. Children whose parents had less than six months of exposure to OPs were excluded from the study. For comparison, we selected at least 56 children from an urban population (Tijuana, B.C.) without pesticide exposure. The study area is justified because San Quintín, Baja California, is one of the main agricultural zones in northwestern Mexico, with intensive production of vegetables and other crops that require the continuous use of OP. Thousands of agricultural workers in this region apply, handle, and come into direct contact with agrochemicals, thereby increasing the risk of occupational exposure. Furthermore, a large portion of the population resides in communities with high social vulnerability, where families often live near fields in conditions that facilitate environmental exposure {Formatting Citation}.

### Ethical statement

This study adhered to the principles of the Declaration of Helsinki and received approval from the Ethics Committee of the Faculty of Medicine and Psychology of the Autonomous University of Baja California (FMP-PI2019-242). Before each interview, informed consent was obtained from the parents through written and verbal explanations detailing the purpose of the study and the estimated duration of the interviews. Participants were also assured they understood their right to withdraw without risk or compensation.

### Data collection

Face-to-face interviews were conducted with farm workers exposed to OPs to assess the food safety of their children. The same method was applied to non-exposed volunteers under identical conditions. Food security was evaluated using the Mexican Food Security Scale (MFSS) (Villagómez-Ornelas et al. 2014), supplemented by a food frequency questionnaire. Information was gathered on parents' sociodemographic status, economic situation, health, and duration of pesticide exposure. Obtaining specific data on the types of pesticides used on farmland was challenging, as farm workers are often unaware of the exact mixtures used for pest management in agricultural fields. However, information regarding pesticide usage was sourced from the Ministry of Agriculture, Livestock, Rural Development, Fisheries and Food Supply's (SAGARPA) Agricultural Technical Agenda, and other official sources (Hernández-Antonio and Hansen 2011; Camarena et al. 2012; INIFAP; SAGARPA 2015; González 2017); This source reports that OPs, such as dichlorvos, acephate, naled, parathion, and malathion, are the most used pesticides in Baja, California. Table 1 shows the toxicological profile of these pesticides and their classification according to the WHO (EPA 2016; FAO and WHO 2019; NPIC 2024).

**Table 1:** Organophosphate pesticides are commonly used in Baja California, México, and are presented in its classification and toxicity profile. World Health Organization (WHO) classification: Class IA, extremely hazardous; Class IB, highly hazardous; Class II, toxic; Class III, moderately toxic

Pesticide name	WHO classification	Toxicity profile (effects of long-term exposure)
Acephate	III	Cholinesterase inhibitor; metabolized to methamidophos; motor and behavioral deficits; neuropathological changes, carcinogenic, and endocrine disruptor
Chlorpyrifos	II	Cholinesterase inhibitor; children exposed to chlorpyrifos show developmental delays and disorders, as well as a higher incidence of attention-deficit/hyperactivity disorder (ADHD)
Dichlorvos	IB	Persistent inhibition of AChE, chronic fatigue, mood changes, impaired memory and concentration, mild neuropathy with prolonged exposure, hormonal changes, reproductive disorders, thyroid disorders, and carcinogens (in animals)
Malathion	III	Sustained inhibition of cholinesterase (neurotoxic risk), effects on reproduction and development (fetal resorption, fetal impairment in animals), alterations in thyroid function (decreased thyroid activity in rats)
Methamidophos	IB	Insecticide and acaricide; cholinesterase inhibitor; delayed neuropathy (nerve damage), neurotoxic effects, possible toxicity to other organs, reproductive risks
Methyl parathion	IA	Changes in mental state (memory deficits, concentration problems, mood swings). Long-term neurobehavioral effects have been observed in children, with alterations in tests of attention, memory, and motor function
Naled	IB	It inhibits cholinesterase and carboxylesterases, altering neuroendocrine signaling. Metabolized to dichlorvos. It affects thyroid homeostasis and thyroid development in animals
Parathion	IA	Sustained reduction of erythrocyte cholinesterase, possible effects on the reproductive system, changes in spermatogenesis, deficit in nerve function, effects on memory and coordination

### Anthropometrics and indices calculations

Growth disorder is any significant deviation from expected growth according to age and sex (2009). In our study, two main categories were included: wasting (weight-for-height < -2 SD) and stunting (height-for-age < -2 SD) (Yani et al. 2023). Using standardized procedures, we collected five anthropometric measurements (height, weight, tricipital folds, mid-upper arm circumference (MUAC), and head circumference) categorized according to World Health Organization (WHO) Child Growth Standards (Ebrahim 2007). According to the WHO criteria for childhood malnutrition, a child is considered to have a growth disorder if their Z-score is less than −2 standard deviations in any of these categories. Anthropometric data were classified and interpreted using growth charts and sex and age-specific cutoff values from the WHO (WHO 2006; Ebrahim 2007, 2009). The Body Mass Index (BMI) was computed using Microsoft Excel spreadsheets as weight divided by height squared (kg/m^2^). Children's height and BMI Z-scores were derived from the WHO Child Growth Standards (2009). Weight was measured using a TANITA® floor scale (model BF689, Arlington Heights, IL) with a resolution of 0.100 kg. Children removed their shoes and outer clothing such as jackets and cardigans. They stood with feet together in the center of the scale, heels against the back edge, arms hanging freely at their sides, and heads facing forward, not downward. Height was measured using a portable SECA 206® stadiometer (Hamburg, Germany) with a range of 0–220 cm and an accuracy of 1 mm. Children removed their shoes and stood with arms hanging freely at their sides, backs straight, heels against a vertical measure, and heads positioned according to the Frankfort plane. They were instructed to look straight ahead and exhale; measurements were taken at the end of exhalation. Head circumference was measured using a non-stretchable tape passed over the eyebrows and ears around the back of the head. We located the midpoint between the acromion (shoulder) and the olecranon (elbow) of the non-dominant arm to obtain the triceps skinfold measurement. With the child standing and the arm relaxed, we measured the skin and fat vertically. The measurement was taken with the Harpenden caliper after 2 s of stabilization.

### Household food security

Food security is the state in which all people, at all times, have physical, social, and economic access to sufficient, safe, and nutritious food to lead an active and healthy life. Its four main dimensions are: availability, access, utilization, and stability (Cfs 2012). Food security was assessed using the MFSS (Villagómez-Ornelas et al. 2014), designed to identify households experiencing severe food insecurity. Its primary goal is to capture household experiences of food insecurity and track changes in food acquisition over the past three months relative to available resources. The scale categorizes households into four groups: food security, mild food insecurity, moderate food insecurity, and severe food insecurity. It evaluates perceived food insecurity experiences over the preceding three months, focusing on distinguishing access to food for adults and individuals under 18. The self-report scale comprises 12 questions arranged by severity, each offering binary responses (yes/no) scored as 1 or 0, respectively. The total score, calculated from affirmative responses, determines the level of food insecurity. The MFSS consists of two sections: an initial set of six questions, with an additional six questions for households with members under 18. For households without minors, scores of 0 indicate food security, 1–2 indicate mild food insecurity, 3–4 indicate moderate food insecurity, and 5–6 indicate severe food insecurity. For households with minors, the thresholds are 1–3 for mild food insecurity, 4–7 for moderate food insecurity, and 8–12 for severe food insecurity (Villagómez-Ornelas et al. 2014; Fierro-Moreno and Lozano-Keymolen 2023; Vargas-Vázquez et al. 2023).

### Sample preparation and clinical biochemistry

Peripheral venous blood samples were taken from all participating children between 7:00 and 9:00 am. The vacutainer system collected 8 mL of venous blood, 3 mL in tubes with K_2_EDTA anticoagulant, and 5 mL in red tubes without additives. The sample in the red tube was separated from the globular package by centrifugation for 10 min at 3000 rpm to obtain 3 mL of serum. All collected samples were immediately processed to determine biochemistry parameters, hormonal markers, complete blood count, and plasmatic BChE with quality control and standards. The quantitative determination of thyroid hormones, Thyrotropin (TSH), free thyroxin (fT4), and total triiodothyronine (T3) was measured by a microplate enzyme immunoassay type 3 (Accubind Elisa microwells, Monobin Inc., USA). The quantitative determination of growth hormone (GH) was measured by a microplate enzyme immunoassay type 3 (Accubind Elisa microwells, Monobin Inc., USA). Insuline-Like Growth Factor 1 (IFG-1) was measured by enzyme-linked immunosorbent assay (Sigma Aldrich, México). Hematology was measured using an automatic analyzer, Zybio 5 (Zybio Inc., Guangdong, China). The clinical biochemistry parameters (Table 6). BChE was measured by colorimetric methods at 37 °C, using an automatic analyzer Mindray BS200 (Mindray Inc., Shenzhen, China) with commercial kits (Wiener lab®, Santa Fe, Argentina, and Pointe Scientific®, Michigan, USA) (ISO 9001; ISO 13485).

### Determination of cholinesterase activity

All the collected samples were processed immediately to determine AChE activity according to the Worek method (Worek et al. 2005). A blood dilution (1:100, prepared with Triton X-100), 1 mL of phosphate buffer (0.1 M, pH 7.4), and 0.05 mL of DTNB (5,5-dithiobis 2-nitrobenzoic acid, 10 mM) were added together; the mixture was incubated at 37 °C for 10 min, and then 0.025 mL of acetylthiocholine iodide (28.3 mM) was added. The absorbance was monitored at 436 nm for 3 min using a UV/Vis spectrophotometer (DU-50 Beckman Coulter). Hemoglobin concentration was determined using a hematology analyzer (Zybio 5). The amount of substrate hydrolyzed was correlated with hemoglobin concentration levels to calculate the AChE-specific activity and was expressed as U/gHb. Triplicate analyses of blood samples assessed the reproducibility of the enzymatic analyses. In each case, the coefficient of variation was 8% or less. The linearity of the Worek method test was taken to determine the activity of AChE (mE/min) and converted to U/L. The linear regression analysis showed a high correlation (r^2^ = 0.9975, y = 52.3536x –1.5994). The AChE activity was standardized for hemoglobin read from hematological analysis performed for each sample (specific activity). BChE was measured by colorimetric methods at 37 °C, using an automatic analyzer Mindray BS200 (Mindray Inc., Shenzhen, China) with commercial kits (Wiener lab®, Santa Fe, Argentina, and Pointe Scientific®, Michigan, USA) (ISO 9001; ISO 13485). The minimum detectable change in BChE activity is 23 U/L.

### Quality control of biochemical measurements

Quality controls were performed to monitor the performance of the hormonal, enzymatic, biochemical, and hematological assays. The precision of the systems was evaluated through multiple runs using control sera to determine the mean value, standard deviation, and coefficient of variation. A significantly lower standard deviation (1 SD) and a CV of 10% than the established mean value indicated the reliability of the assays.

### Data analysis

SPSS software package 21.0 (Chicago, IL) and GraphPad Prism Project 10.0 (San Diego, CA) were used for all statistical analyses. Descriptive statistics included mean, median, standard deviations, and range values for continuous data; categorical data included percentages and frequencies. Comparisons between groups were analyzed using the Student's t-test, the Chi-square test, and ANOVA, depending upon the distribution of the variableand simple multivariable linear regression was used to assess the relationship of quantitative variables, and simple linear regression adjusting for gender and age. Factorial ANOVA was used to examine multiple categorical factors simultaneously and to detect interaction effects.

## Results

### Children's anthropometric characteristics

One hundred and ninety children were studied, of which 134(70.52%) were in the indirectly exposed group. That group is children with parents who have direct pesticide exposure and live in agricultural fields. The non-exposed group consisted of children living in the city (56, 29.47%), taken as controls for the present study. The results regarding the anthropometric measurements of the children are shown in Table 2, in which we can observe several statistically significant differences (*p* < 0.001) in terms of weight, height, MUAC, head circumference, and BMI.

**Table 2:** Anthropometric variables between children nonexposed and children indirectly exposed to pesticide contamination. Data on age, gender, and education are presented as frequencies (N) and percentages (%)

General variables	Non-exposed (n = 56)	Indirectly Exposed (n = 134)	*p*
	(N, %)	(N, %)	
*Age (Years) (N, %)*			< 0.001
2—3	9 (16.1%)	25 (18.7%)	
4—5	16 (28.6%)	70 (52.2%)	
6—7	17 (30.4%)	35 (26.1%)	
8—9	14 (25%)	4(3.0%)	
*Gender (N, %)*			0.534
Female	28 (51.8%)	62 (46.2%)	
Male	27 (48.2%)	72 (53.7%)	
*Education (N, %)*			< 0.001
Playgroup	18 (38.3%)	100 (76.9%)	
Elementary school	29 (61.7%)	24 (18.5%)	
None	0(0%)	6 (4.6%)	
*Weight (Kg)*			< 0.001
Female	22.35 (7.86)	18.11 (2.99)	0.515**
Male	24.20 (8.82)	17.72 (3.38)	0.197***
*Height (cm)*			< 0.001*
Female	114.86 (11.86)	106.93 (9.39)	0.340**
Male	112.76 (13.93)	105.20 (10.19)	0.914***
*Head circumference (cm)*^a^			< 0.001*
Female	51.38 (1.86)	47.64 (4.87)	0.040**
Male	51.74 (2.89)	48.74 (2.37)	0.579***
*Tricipital folds (mm)*			0.059*
Female	9.41 (3.69)	9.76 (1.83)	0.352**
Male	10.11 (3.39)	8.27 (1.88)	0.0024***
*MUAC (cm)*			< 0.001
Female	19.83 (3.60)	16.40 (4.56)	0.949**
Male	20.41 (3.32)	15.69 (1.45)	0.275***
*BMI (kg*^*2*^*/cm)*			< 0.001
Female	16.60 (2.99)	15.83 (1.61)	0.247**
Male	18.30 (3.39)	15.93 (1.18)	0.028***

^a^The head circumference was determined for children up to 5 years of age according to the WHO (WHO 2006; Ebrahim 2007). MUAC (Midd Upper Arm Circumference), BMI (Body Mass Index). The differences were tested using Pearson´s Chi-square statistic for categorical variables, and a factorial ANOVA was used to evaluate differences in continuous dependent variables, which were considered significant at *p* < 0.05

*Significance for group; **significance for gender; ***Significance for gender and group interaction

The indirectly exposed group showed a significantly lower weight compared to the control group (4.24 kg less in girls and 6.48 kg less in boys), (F_(1,188)_ = 39.80, *p* < 0.001), and showing no difference between genders or interaction. In terms of height, a shorter length is also observed in the group of indirectly exposed children compared to the control group (F_(1,188)=_21.33, *p* < 0.001), (7.93 cm less in girls and 7.56 cm less in boys); no significant differences were observed between genders. With these results, it can be determined that the group exposed indirectly is smaller than the control group, which is confirmed by a significant difference in the BMI results (F_(1,188)_ = 22.02, *p* < 0.001). Regarding head circumference, a smaller circumference is also observed in the indirectly exposed group compared to the control (3.74 cm less in girls and 3 cm less in boys), (F_(1,188)_ = 52.23, *p* < 0.001), significant difference was also observed between girls and boys, suggesting that head circumference differs between the groups (F_(1,188)_ = 4.247, *p* = 0.0407). These results suggest that pesticide exposure has a significant effect on head growth, while gender differences are also present, with no evidence of interaction between the two factors.

MUAC showed less circumference in indirectly exposed children (3.74 cm less in girls and 4.72 cm less in boys) compared to the control group (F_(1,188)_= 60.84, *p* < 0.001). The data presented above shows that children indirectly exposed to pesticides are smaller than the control group (non-exposed) there being no differences between genders.Finally, in the tricipital folds, no significant differences were observed between groups or between genders; however, a significant interaction was identified (*p* = 0.0024), which indicates that environmental exposure affects boys and girls differently.

Once we determined the anthropometric measurements in Table 2, we interpreted and diagnosed the calculated anthropometric indices. BMI was calculated as weight/height (kg/m^2^). The children’s height and BMI Z-scores were calculated based on the WHO Child Growth Standards (WHO 2006; Ebrahim 2007) and were categorized as being at risk of being normal, overweight, underweight, and obese, respectively. Stunting was defined as height-for-age Z-score + 1, >  + 2, and >  + 3, which was categorized as being at risk of being normal, low, slightly tall, and slightly low. For the present study, we defined ‘concurrent stunting and overweight’ as children with a combination of height-for-age Z-score + 1. Tricipital folds and arm circumference Z-score + 1, −2, −3 were categorized as normal, moderate malnutrition, malnutrition risk, overweight, and obese, respectively. The World Health Organization (WHO 2006; Ebrahim 2007) used the growth tables and cut-off values by sex and age. The interpretation or diagnosis of the anthropometric data is shown in Table 3.

**Table 3:** Anthropometric indices between children nonexposed and children indirectly exposed to pesticide contamination. Data are presented as a comparison of percentages (%) between groups. The differences were tested using Pearson´s Chi-square, *p* < 0.05

Variable (diagnosis)	Non-exposure	Indirectly exposure	P
*Weight for age*			< 0.001
Normal	58.2	70.7	
Mild malnutrition	12.7	11.3	
Overweight	7.3	15.8	
Obese	20	2.3	
Malnutrition	1.8	0	
*Height for age*			0.023
Low	16.4	18	
Normal	45.5	50.4	
Slightly tall	18.2	4.5	
Slightly low	20	27.1	
*Weight for height*			< 0.001
Normal	58.2	70.8	
Mild malnutrition	12.7	11.2	
Overweight	7.2	15.7	
Obese	20	2.2	
Malnutrition	1.8	0	
*Head circumference*^a^			0.006
Normal	100	74.7	
Microcephaly	0	25.3	
*Tricipital folds*			< 0.001
Normal	52.7	80.4	
Malnutrition risk	10.9	3.1	
Moderate malnutrition	7.3	2.1	
Overweight risk	5.5	12.4	
Overweight	0	2	
Obese	23.6	0	
*Arm circumference*			< 0.001
Normal	66.7	72.2	
Moderate malnutrition	0	4.4	
Malnutrition risk	0	23.3	
Overweight	16.7	0	
Obese	16.6	0	
*Body mass index*			0.012
Normal	65.5	69.2	
Underweight	1.8	6.8	
Overweight	14.5	19.5	
Obese	18.2	4.5	

Significant differences were shown between groups in all variables. Regarding weight for age, which reflects the weight relative to the child's age, malnutrition was not observed in the indirectly exposed group; most children had a normal weight for age. (X^2^(4) = 21.968, *p* < 0.001).

Stunting (height for age) is defined as a condition of a child with less than expected height (Yani et al. 2023). The group of indirectly exposed children showed a higher percentage of low height for age than the control group, followed by slightly lower. However, 50% of the children were of normal height. In the category of slightly tall height for age, a higher percentage was presented in the control children than in the indirectly exposed children (18.2% and 4.5%, respectively) (X^2^(3) = 9.577, *p* = 0.023).

From the head circumference result, we can observe that a significant percentage (25.3%) of indirectly exposed children fall into the microcephaly category associated with developmental delay (Rau et al. 2021). Regarding tricipital folds and arm circumference, there are significant differences between groups X^2^(5) = 35.620, *p* = 0.000 and X^2^(4) = 34.749, *p* < 0.001. No data on obesity was observed in the group of indirectly exposed children. A higher percentage of normal values was observed in indirectly exposed children compared to control children in both variables, and low percentages of moderate malnutrition in both groups and both variables. Finally, both groups were categorized with a high percentage of normal BMI (65.5% for the controls and 69.2% for the indirectly exposed group). Low weight was present in 6.8% of the indirectly exposed children compared to the control children (1.8%).

On the other hand, we also studied the sociodemographic factors of the parents of both groups of children. Table 4 shows the data collected regarding the study of the sociodemographic status, health, and time of exposure to pesticides that the parents have been exposed to pesticides. The parents of the non-exposed children (non-exposed parents) had an average age of 32.4 years, with 62.9% female and 37.1% male. The average age of the parents of the indirectly exposed children (exposed parents) was 23.4 years, with 60.0% being female and 40% male. Among the parents of indirectly exposed children, 55.7% had primary education, 27.1% had secondary education, and the highest percentage of parents of non-exposed children had middle school education (97%). No significant differences were observed in the sociodemographic variables between the exposed and non-exposed groups of parents, except for the education and pesticide exposure time variables (*p* < 0.001 and *p* = 0.001, respectively).

**Table 4:** The parent´s sociodemographic status, economics, health, and pesticide exposure time

Variable	Non-exposure	Exposure	*p*
	Parents	parents	
	N (%)		
*Education*			< 0.001
Illiterate	1 (1.8)	7 (5.4)	
Elementary school	6 (10.7)	68 (52.3)	
Middle school	12 (21.4)	47 (36.2)	
High school	23 (41.1)	8 (6.2%)	
College	14 (25)		
*Marital status*			0.116
Single	9 (16.1)	11 (8.5)	
Free union	24 (42.9)	64 (49.2)	
Married	19 (33.9)	51 (32.9)	
Divorced	2 (3.6)	4 (3.1)	
Widowed	0	2 (3.6)	
Pesticide exposition time (years)^a^	0	5.37 ± 5.16	0.001
Smokers	16(28.6)	16 (12.3)	0.835
*Abuse substances*			0.597
Cocaine		1(0.7)	
Marihuana	1 (1.8)	3 (2.2)	
Cristal	1 (1.8)	1 (0.7)	
No abuse substances	54 (96.4)	129 (96.3)	

^a^Data on exposure time is represented as mean and standard deviation

Here, the most significant data was for the time of pesticide exposure, determined by the time the parents worked in the agricultural fields; the average time was 5.37 ± 5.16 years. However, the data regarding pesticide use was taken from the Ministry of Agriculture, Livestock, Rural Development, Fisheries, and Food Supply’s (SAGARPA, by its initials in Spanish) Agricultural Technical Agenda (Hernández-Antonio and Hansen 2011; INIFAP; SAGARPA 2015; González 2017).

### Food security study

Household food security influences the nutritional status of its members (Kehinde and Favour 2020) and was measured using the MFSS (Fierro-Moreno and Lozano-Keymolen 2023); the descriptive analysis is presented in Table 5. Here, it is revealed that food safety is presented with a high prevalence in both groups studied, 48.21 and 57.46%, the indirectly exposed group being the one with the highest percentage in food safety, or there is no decrease in quantity and quality of food (Vega-Macedo et al. 2014). Mild food insecurity implies a reduced food quality observed in both groups studied, with similar statistics. Moderate food insecurity implies a reduced quality and quantity of food, observed in a low percentage (11.19%) of indirectly exposed children. The most severe data on severe household food insecurity occurs in the control group at 8.92%, which indicates that in households with children under 18 years of age, the quality and quantity of the food consumed decreased (Villagómez-Ornelas et al. 2014; Fierro-Moreno and Lozano-Keymolen 2023).

**Table 5:** Nutritional status of 2–9-year-old children in food-secured households of northwestern México. The data were analyzed using chi-square(X^2^) for group differences

Variable	Non-exposed (n = 56)	Indirectly exposed (n = 134)	*X*^*2*^
	N, (%)	N, (%)	
*Food security classification*			1.883^a^
Food security	27 (48.21)	77 (57.46)	
Mild food insecurity	15 (26.78)	34 (25.37)	
Moderate food insecurity	9 (16.07)	15 (11.19)	
Severe food insecurity	5 (8.92)	8 (5.97)	

^a^According to the statistical analysis, no significant difference exists between groups. (*p* = 0.597)

The data presented are organized in a contingency table (Table 5) that shows the frequency and percentage of participants in each food security category for both groups. To perform the statistical analysis, the chi-square test was considered to evaluate whether there is a significant association between the degree of exposure and food safety between groups, in which it is shown that said relationship does not exist (X^2^ = 1.88, *p* = 0.597). The analysis of household food security revealed that although the majority of families in both groups reported an adequate level of food security, with minimal differences in mild and moderate insecurity, this indicates that the growth problems identified in children (Tables 2 and 3) are not directly linked to the availability or quality of food in the home.

### Clinical biochemistry

Clinical biochemistry was analyzed in all participating children to assess their nutritional and health status once anthropometric measurements and food safety were obtained. In addition to evaluating nutritional status, we determined liver and kidney function. We used the reference values provided by the Wiener Lab (Argentina) and Pointe Scientific (USA) reagent manufacturers to determine whether the values obtained for each analyte were within the average high or low range. Overall, the results demonstrate that both groups are in optimal condition regarding clinical biochemistry. Parameters related to the nutritional status of the children, such as albumin, creatinine, total proteins, iron, phosphorus, and calcium, were taken into account. The results are presented in Table 6, showing no significant differences in total proteins, creatinine, and iron between the non-exposed group and the indirectly exposed group (*p* = 0.96, *p* = 0.243, *p* = 0.728, respectively). Regarding calcium, a slightly higher result is observed in the indirectly exposed group (9.99 ± 0.63 mg/dL) compared to the non-exposed group, where 7.14% of the samples obtained a result below the normal range (*p* = 0.04). In the average albumin level, a higher value is observed in the indirectly exposed group (4.20 g/dL, *p* < 0.001) compared to the non-exposed group, where 7.15% of the samples had a result below the normal range. The average albumin level is higher in the indirectly exposed group (4.20 g/dL, *p* < 0.001) than in the non-exposed group, as 7.15% of the samples had a result below the normal range. The renal function of the participating children was analyzed, considering the previously described parameters and nitrogen metabolism (ureic nitrogen), whose average falls within typical values for both groups. However, a significant difference exists between the two groups (*p* < 0.001), as the indirectly exposed group shows a slightly higher average of the analyte (23.11 ± 5.71). Total, bilirubin, and the liver enzymes Aspartate aminotransferase (AST), Alanine aminotransferase (ALT), Gamma-glutamyl transferase (GGT), and alkaline phosphatase (ALP) were considered to evaluate liver function. Significant differences are observed between both groups for all analytes (*p* < 0.001), except for ALT (*p* = 0.611). This is evident in total bilirubin, which shows a lower average (0.14 mg/dL) in the indirectly exposed group compared to the non-exposed group (0.61 mg/dL), as 5% of the samples in this group had a value higher than the normal range. Regarding AST and GGT, significant differences are observed between both groups (*p* < 0.001) because, in both enzymes, the indirectly exposed group showed a higher average, as 6% of the samples had a value above the normal range for both enzymes. An interesting observation is noted in ALP because the average of the analyte is 1.5 times lower in children indirectly exposed compared to non-exposed children, yielding a statistically significant difference (*p* < 0.001). Only 21% of indirectly exposed children and 92% of non-exposed children obtained values above the normal range. This result suggests an increased osteoblastic activity in non-exposed children, which is typical for their full development and growth. Only cholesterol and triglycerides were considered in the clinical biochemistry results to assess the lipid profile. Most children in both groups are within normal ranges with no significant differences (*p* = 0.487 and *p* = 0.125, respectively) and without clinical significance. These results show that both groups of children had parameters within normal ranges, reflecting adequate nutritional and health status. However, significant differences in some markers, particularly alkaline phosphatase and liver enzyme abnormalities in the indirectly exposed children, suggest possible effects of environmental pesticide exposure on bone growth and liver function, even without obvious malnutrition.

**Table 6:** Effects on biochemistry profiles in children’s groups, indirectly exposed and non-exposed (controls). Data are expressed as the mean ± SD; each parameter includes a reference range

Parameter (Reference range)	Non-exposed (n = 56)	Indirectly Exposed (n = 134)	*t*	*gl*	*p*
	(Mean ± SD), %	(Mean), %			
*Cholesterol (mg/dl)*	(147.68 ± 49.36)	(142.47 ± 25.26)	0.698	109	0.487
Normal (< 200)	94.65	99.25			
High	5.35	0.75			
*Triglycerides (mg/L)*	(112.02 ± 22)	(124.93 ± 53.05)	9.00	83.77	0.125
Normal (< 150)	100	87.31			
High	0	12.68			
*Creatinine (mg/dl)*	(0.42 ± 0.23)	(0.38 ± 0.07)	− 1.01	64.81	0.243
Normal (0.04–1.4)	100	100			
*Ureic nitrogen (mg/dl)*	(20.07 ± 3.12)	(23.11 ± 5.71)	15.90	83.74	< 0.001
Normal (4.7–23.4)	100	100			
*Total protein (g/dL)*	(7.07 ± 1.44)	(7.04 ± 0.59)	− 0.93	73.12	0.926
Normal (6.1–7.9)	12.5	97.76			
Low	5.35	2.23			
High	7.15	0			
*Albumin (g/dL)*	(3.78 ± 0.81)	(4.20 ± 0.25)	3.70	65.60	< 0.001
Normal (3.5–4.8)	92.85	100			
Low	7.15	0			
*Total bilirubin (mg/dL)*	(0.61 ± 0.31)	(0.14 ± 0.08)	− 10.6	62.88	< 0.001
Normal (0.2–1.2)	94.65	100			
High	5.35	0			
*AST (UI/L)*	(26.41 ± 8.93)	(33.75 ± 9.41)	4.21	109	< 0.001
Normal (< 40)	98.22	94			
High	1.78	6			
*ALT (UI/L)*	(16.19 ± 8.88)	(15.5 ± 5.04)	− 0.51	109	0.611
Normal (< 38)	96.43	100			
High	3.57	0			
*GGT (UI/L)*	(11.33 ± 3.91)	(20.8 ± 7.77)	8.03	79.47	< 0.001
Normal (8–54)	100	93.28			
High	0	6.72			
*ALP (UI/L)*	(615.39 ± 173)	(404.87 ± 12.65)	− 8.12	84.97	< 0.001
Normal (100–400)	7.14	78.35			
High	92.85	21.64			
*Phosphorous (mg/dL)*	(4.93 ± 0.5)	(5.72 ± 0.98)	5.23	80.72	< 0.001
Normal (4–7)	100	100			
Low	0	0			
High	0	0			
*Calcium (mg/dL)*	(9.25 ± 2.52)	(9.99 ± 0.63)	2.08	61.93	0.041
Normal (8.5–10.5)	75	94			
Low	7.14	1.5			
High	17.86	4.5			
*Iron (μg/dL)*	(76.41 ± 32)	(78.61 ± 34.22)	0.349	10.09	0.728
Normal (50–175)	82.14	88.8			
Low	17.86				
High	0				

AST, Aspartate aminotransferase; ALT, Alanine aminotransferase; GGT, Gamma-Glutamyl Transferase; ALP, Alkaline phosphatase

In Table 7, the results of the hormonal profile are shown. The averages show a 2.55 and 1.28 times decrease in GH and IFG-1, respectively, in the directly exposed children compared to the control children. This indicates that although exposure has a measurable effect, it has not translated into clinically deficient levels. However, the averages fall above normal values. For thyroid hormones, an increase is observed in general: 1.90 and 5.24 times for fT4 and tT3, respectively. The average quantification of these hormones is slightly out of the normal range in indirectly exposed children; this could indicate a stimulation or alteration of the thyroid axis in this group. TSH does not show significant differences (*p* = 0.557), suggesting that the hypothalamic-pituitary-thyroid axis may maintain homeostasis despite changes in peripheral hormones. No significant differences were found between boys and girls on any parameter, nor was there any interaction between group and gender. This indicates that the observed effects are consistent regardless of gender.

**Table 7:** Effects on hormonal profiles in children groups indirectly exposed and in non-exposed (controls)

Hormone (Expected range of values)	Non-exposed (n = 56)	Indirectly Exposed (n = 134)	*F*	*p*
	Mean ± SD	Mean ± SD		
*hGH, μIU/ml (0–55)*	8.71 ± 2.5	3.41 ± 1.33	5.64	0.018*
Female	11.33 ± 3.2	3.21 ± 0.94	1.34	0.248**
Male	5.88 ± 1.5	3.57 ± 1.58	1.75	0.188***
*IGF-1, ng/ml (34–226)*	70.79 ± 58.92	55.14 ± 39.11	4.39	0.037*
Female	74.63 ± 59.80	58.39 ± 42.67	0.92	0.338**
Male	66.65 ± 58.81	52.38 ± 35.78	0.018	0.893***
*TSH, μIU/ml (0.28–6.82)*	3.12 ± 1.8	3.38 ± 3.0	0.346	0.557*
Female	2.93 ± 1.77	3.50 ± 3.6	0.040	0.843**
Male	3.33 ± 1.8	3.27 ± 2.4	0.537	0.465***
*fT4, ng/dl (0.8–2.0)*	1.22 ± 0.22	2.33 ± 0.65	150.74	< 0.001*
Female	1.22 ± 0.21	2.37 ± 0.54	0.181	0.671**
Male	1.23 ± 0.23	2.29 ± 0.74	0.208	0.649***
*tT3, ng/ml (0.52–1.85)*	0.65 ± 0.59	3.41 ± 1.33	218.71	< 0.001*
Female	0.74 ± 0.55	3.21 ± 0.94	0.242	0.623**
Male	0.56 ± 0.63	3.57 ± 1.59	2.11	0.147***

Data are expressed as the mean ± SD., Anova Factorial. Each parameter includes a reference range for each parameter. * significance for group; **significance for gender; ***Significance for gender and group interaction.

Growth hormone (hGH), range: 0–55μUI/ml, Insulin-Like Growth Factor 1(IFG-1), range: 34–226 ng/ml, Thyrotropin (TSH), range: 0.28–6.82μUI/ml, Free thyroxin (fT4), range 0.8–2.0 ng/dl**,** Total triiodothyronine (tT3), range 0.52–1.85 ng/ml

### Determination of AChE and BChE activity

The activity of AChE and BuChE (Table 8) was used in this study as valuable biomarkers of the effect of environmental exposure to OPs (Dalmolin et al. 2020). The mean activity of AChE was 7211 ± 1917 and 8368 ± 213(U/L) in the indirectly exposed and control groups, respectively [F_(1,186)_ = 19.87, *p* < 0.001]. Still, the groups have no significant differences when divided according to gender. The specific activity does demonstrate significant differences between groups [F_(1,186)_ = 10.07, *p* = 0.002], too, except for genders (*p* = 0.671); moreover, there are significant differences between groups when hemoglobin values are analyzed [F_(1,186)_ = 21.692, *p* < 0.001].

The study demonstrated a significantly lower activity into plasmatic cholinesterase (BChE), which is sometimes more strongly inhibited than AChE (Lionetto et al. 2013), and is observed in our results; the mean activity of BChE was 6426 ± 1664 and 8025 ± 1462(U/l) [F_(1,186)=_42.202, *p* < 0.001] in the indirectly exposed and control groups, respectively, similar differences were observed between gender [F_(1, 186)_ = 7.6713, *p* = 0.006]. The BChE (U/l) values by sex indicate that boys have higher values (7179.78 ± 1797.04) than girls (6593.35 ± 1681.37), regardless of their group. To determine whether BChE levels increase with age, a linear regression was performed on the samples of the 54 control children. A positive slope (45.14) was obtained, suggesting a slight increase in BChE with age, estimated at 45.14 units per year. However, the 95% confidence interval for the slope (− 165.8 to 256.1) included the null value, and the statistical analysis was not significant (*p* = 0.669, F = 0.1841). This indicates that, in this sample, it cannot be stated that BChE levels increase significantly with age. However, it should be considered that the moderate sample size (n = 54) limits the statistical power of the análisis.

These results demonstrate that environmental exposure to organophosphate pesticides in children is associated with significant inhibition of cholinesterases, particularly BChE, which is confirmed as a sensitive biomarker of exposure.

**Table 8:** Influence of gender on the activity of the AChE and BuChE enzymes in the studied populations

Parameter	Nonexposed Mean (SD)	Indirectly Exposed Mean (SD)	p
*Hb (g/dL)*	12.99 (1.25)	12.28 (0.84)	< 0.001*
Female	12.88 (1.41)	12.11 (0.67)	0.092**
Male	13.10 (1.05)	12.42 (0.94)	0.768***
*Specific AChE (U/g Hb)*	65.34 (10.14)	58.39 (14.74)	0.002*
Female	66.42 (12.48)	59.13 (14.17)	0.414**
Male	64.04 (6.20)	56.81 (12.46)	0.826***
*AChE Activity (U/L)*	8368.12 (213.06)	7211.02 (1917.81)	< 0.001*
Female	8387.58 (175.88)	7262.10 (1644.36)	0.815**
Male	8342.23 (240.80)	6972.81 (1545.24)	0.938***
*BChE (U/L)*	8025.66 (1462.71)	6426.79 (1664.88)	0.000*
Female	7651.93 (1677.41)	6090.09 (1442.65)	0.006**
Male	8427.07 (1082.89)	6712.05 (1792.91)	0.762***

Data are expressed as the mean ± SD. Anova Factorial. * significance for group; **significance for gender; ***Significance for gender and group interaction.

Hemoglobin (Hb), reference values female: 11.40 to 16.10 g/dL, male: 11.60 to 16.00 g/dL (Piedra et al. 2012). Specific activity of acetylcholinesterase (Specific AChE). Acetylcholinesterase (AChE). Butirylcholinesterase (BChE), reference values 5320–12920 U/l (Wiener Lab).

### Association between biomarkers, exposure, and anthropometric measurements.

A multivariable linear regression analysis (Table 9) assessed the relationship between environmental factors (AChE, BChE, parental exposure time), biomarkers, and the anthropometric measurements of the participating children (indirectly exposed group). BChE showed a negative correlation with GGT (β = − 43.47, 95% IC = − 84.87 to − 2.075, *p* = 0.039), indicating hepatic stress. No associations were found between BChE levels, the other biomarkers, and the anthropometric measurements analyzed.

**Table 9:** Multiple linear regression model

Variable	BChE			AChE			Parental exposure time		
	β, 95%IC, p			β, 95%IC, p			β, 95%IC, p		
Age	− 157.6	− 374.3 to 59.13	0.152	235.7	− 200.5 to 671.9	0.286	− 0.342	− 1.46 to 0.77	0.545
Gender	398.7	− 200.3 to 997.7	0.190	192.1	− 535.5 to 919.7	0.601	− 0.541	− 2.40 to 1.31	0.565
Height	19.69	− 52.79 to 92.17	0.591	0.057	− 0.157 to 0.27	0.959	0.057	− 0.15 to 0.27	0.595
Weight	113.4	− 121.1 to 348.0	0.339	0.068	− 0.619 to 0.75	0.843	0.068	− 0.61 to 0.75	0.843
AChE	− 0.091	− 0.244 to 0.084	0.338				0.0004	− 0.00087 to 5.7e− 005	0.085
BChE				− 0.107	− 151.0 to − 9.63	0.022	0.0003	0.0009071 to 0.0001	0.200
Exposition time	− 49.34	− 113.3 to 14.67	0.129	− 80.31	− 67.83 to 139.7	0.059			
Hb	− 94.30	− 482.7 to 294.1	0.631	− 344.2	− 768.4 to 80.04	0.110	− 0.790	− 1.89 to 0.30	0.157
hHG	0.366	− 232.4 to 233.2	0.997	− 171.5	− 438.6 to 95.48	0.205	0.211	− 0.46 to 0.89	0.538
IFG-1	0.468	− 7.690 to 8.62	0.909	− 5.21	− 14.59 to 4.161	0.272	0.0072	− 0.0166 to 0.031	0.549
TSH	− 4.55	− 93.49 to 84.38	0.919	43.71	− 58.72 to 146.1	0.887	− 0.249	− 0.504 to 0.0062	0.055
fT4	− 49.16	− 539.2 to 440.9	0.842	− 206.5	− 771.5 to 358.5	0.470	0.994	− 0.42 to 2.41	0.166
t3T	− 59.83	− 189.6 to 69.98	0.363	− 2.20	− 148.4 to 152.8	0.467	− 0.383	− 0.72 to − 0.045	0.026
Albumin	− 463.9	− 2516 to 1588	0.654	524.8	− 1846 to 2896	0.661	2.289	− 3.59 to 8.16	0.442
ALP	− 2.91	− 7.721 to 1.887	0.231	0.280	− 1846 to 2896	0.921	0.022	− 0.0356 to 0.009	0.001
AST	3.928	− 34.17 to 42.03	0.838	− 13.72	− 57.68 to 30.24	0.537	0.0422	− 0.152 to 0.068	0.450
Calcium	783.3	− 87.69 to 1654	0.077	136.5	− 1157 to 884.4	0.791	1.315	− 1.24 to 3.87	0.310
GGT	− 50.23	− 93.66 to − 6.79	0.023	33.04	− 17.97 to 84.04	0.202	0.035	− 0.163 to 0.09	0.583
Phosphorus	− 173.1	− 554.7 to 208.6	0.370	− 244.5	− 684.8 to 195.7	0.273	0.088	− 1.005 to 1.18	0.872
Total bilirubin	1711	− 3064 to 6486	0.479	− 1877	− 7396 to 3641	0.501	1.248	− 12.41 to 14.91	0.856
Ureic nitrogen	− 43.50	− 106.1 to 19.15	0.071	32.94	− 39.80 to 105.7	0.371	0.061	− 0.1218 to 0.24	0.507

Regression coefficients (β) are presented along with their 95% confidence intervals (95% CI). All models were adjusted for gender and age. In the model corresponding to parental exposure time, the adjustment also included parental tobacco, alcohol, and drug use. A *p*-value < 0.050 was considered statistically significant

R^2^(Exposition time) = 0.209, R^2^(AChE) = 0.106, R^2^(BChE) = 0.156

**Fig. 1:**
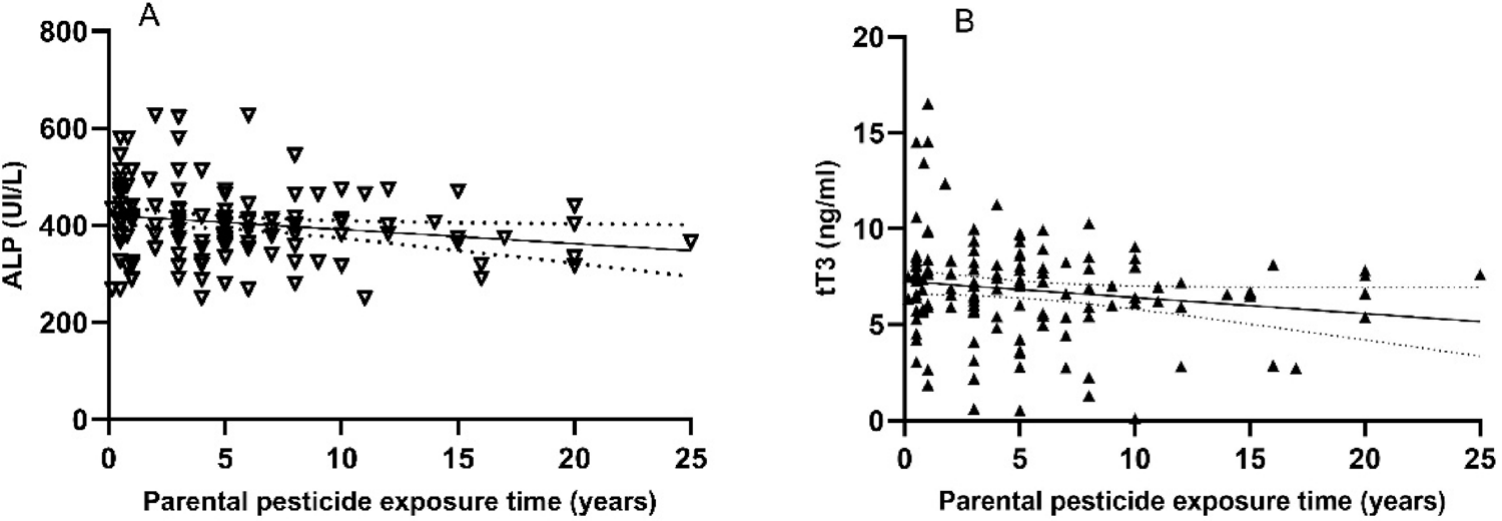
Association between parental exposure time to pesticides and ALP (**A**) and tT3 (**B**) levels. Each panel shows the fitted trend line obtained by multiple linear regression

The association between parental pesticide exposure time and all biomarkers was also examined; the most notable correlation was with ALP (Table 9, Fig. 1A). This analysis revealed a statistically significant negative correlation with ALP (β = − 0.210, 95% IC = –0.036 to –0.009, *p* = 0.001). This association indicates that the longer parents' exposure to pesticides, the lower the ALP levels in children, which can interfere with development and growth. A negative correlation was also observed between parental pesticide exposure time and tT3 (β = − 0.383, 95% IC = − 0.7217 to − 0.04528, *p* = 0.026), although a positive trend was initially expected, the adjusted model suggests that prolonged exposure may be associated with a reduction in the thyroid profile (Table 9, Fig. 1B). No associations were found between cholinesterase levels, the other biomarkers, and the anthropometric measurements analyzed. Regarding the analysis with AChE, no correlation was observed in any of the variables studied; except for BChE (β = − 107, 95% = − 151.0 to − 9.636, *p* = 0.022).

In addition to environmental factors, correlations between anthropometric measurements and some biomarkers were evaluated. Regarding stunting, a positive association was observed with ALP (r = 0.162, *p* = 0.031), which could reflect alterations in bone metabolism. At the same time, a negative correlation was found with IFG-1 (r = − 0.194, *p* = 0.012) and arm circumference (r = − 0.182, *p* = 0.018), indicating that growth retardation is accompanied by lower hormonal levels related to somatic development and a reduction in body mass. Several associations were identified between weight-for-age and several biomarkers. Negative correlation were observed with BUN (r = − 0.209, *p* = 0.008), calcium (r = − 0.152, *p* = 0.04), and IFG-1(r = 0.089, *p* = 0.016), while positive correlation were found with HG (r = 0.089, *p* = 0.016) and T3 (r = 0.164, *p* = 0.030). Negative correlations were also observed among MCHC (r = − 0.169, *p* = 0.026), tricipital folds (r = − 0.249, *p* = 0.002), and BMI (r = − 0.673, *p* < 0.001). These results show that weight-for-age reflects nutritional status and is associated with changes in key metabolic and hormonal markers of child growth.

Finally, the triceps fold showed a negative association with HG (r = − 0.215, *p* = 0.006), which could reflect an inverse relationship between nutritional status and endocrine regulation of growth in the child population evaluated. No associations were found between the anthropometric indices of indirectly exposed children and environmental factors.

## Discussion

OPs are highly toxic to living organisms and their environment, yet they are essential for developing agricultura (Mulla et al. 2020). Farmers are directly exposed to these agrochemicals when working in the fields (De-Assis et al. 2021). People living in agricultural areas may be exposed to higher levels of pesticides than in other regions due to pesticides reaching their homes by pesticide drift or by family members (Norkaew et al. 2013; Nascimento et al. 2018). Farmers' children are potentially exposed to pesticides indirectly via the take-home pathway (Curwin et al. 2007). The analysis of sociodemographic factors shows that the main differences between exposed and unexposed parents lie in educational level and occupational exposure to pesticides. Younger parents with less education tend to be more involved in risky agricultural activities, which increases the likelihood of indirect exposure of their children. This underscores the importance of addressing not only working conditions in the fields but also social determinants, such as education, that can maintain the vulnerability of these families. Our study found that parents of indirectly exposed children have worked in the fields for an average of 5.37 years. This implies that 70.9% of children aged 2 to 5 have been indirectly exposed to OPs. This prolonged exposure, with its potential health risks, indirectly underscores the gravity for workers and their children indirectly (Ádám et al. 2024).

Our data indicate that children in the indirect exposure group show significantly lower weight, height, head circumference, and MUAC values than those in the control group (*p* < 0.001). This suggests that indirect exposure, defined as living in agricultural areas with parents directly exposed to pesticides, may be associated with reduced physical growth (Kartini et al. 2019). We also noted that, on average, exposed girls weighed 4.24 kg less and exposed boys 6.48 kg less compared to the control group, with a height difference of approximately 7.5 cm between the sexes, further reinforcing this result. Although most exposed children have a weight within the expected range for their age, a higher percentage exhibit stunted growth (defined as height-for-age) and microcephaly, raising concerns about their physical and neurological development. Microcephaly affects approximately 1.6/1000 live births and is often associated with developmental delay (Rau et al. 2021). Other common comorbidities include epilepsy, cerebral palsy, and intellectual disability; causes include genetic syndromes, environmental toxins, infectious diseases, and structural brain disorders (Rau et al. 2021). Normal child growth depends on various factors, including genetic, hormonal, and nutritional influences. According to the WHO Multicentre Growth Reference Study (MGRS), infants and young children up to 6 years of age have the potential to grow similarly, regardless of their ethnic origin, as long as they receive adequate nutrition (WHO 2006).

Food security is an essential aspect of the nutritional factor, defined as the condition in which people have, at all times, physical and economic access to sufficient, safe, and nutritious food, enabling them to satisfy their nutritional needs and lead a healthy and active life (Vega-Macedo et al. 2014; Kartini et al. 2019). Household food security refers to a household's ability to meet its members' food needs through production or purchases. Household food security significantly influences the nutritional status of its members (Kehinde and Favour 2020). Our findings suggest a relationship between food security and nutritional status. Most households have adequate access to food, highlighting the importance of ensuring a supply of nutritious options to improve the overall health of children and their families. The high prevalence of food security in both groups studied (48.21% in one group and 57.46% in the other) indicates that, despite exposure differences, many households maintain an acceptable level of food access. The assessment of the association between the degree of exposure and food security suggests that, despite the observed differences, there is no significant relationship (X^2^ = 1.88, *p* = 0.597). This indicates that the growth problems identified in children are not directly linked to the household's availability or quality of food. Therefore, it is suggested that environmental and sociodemographic factors, rather than food security, play a decisive role in the observed differences in child development. Food security is a necessary but insufficient condition to ensure optimal growth in exposed children. Growth retardation and microcephaly appear to be associated with a combination of environmental exposure to pesticides and parental sociodemographic disadvantages, rather than with food availability, underscoring the need for public health policies that integrate both nutritional surveillance and protection against environmental risks. There is abundant recent evidence on the effects of pesticide exposure on somatic growth and neurodevelopment in children (Buralli et al. 2023). Studies on the relationship between organophosphates and fetal growth are still scarce. However, Medley et al. (2025) reported that prenatal exposure to these compounds is associated with alterations in fetal size, as evidenced by elevated concentrations of organophosphate metabolites in maternal urine. They found a sex-dependent pattern: in female fetuses, the negative association with growth is observed in mid-gestation, while in male fetuses, the effects manifest mainly toward the end of pregnancy (Medley et al. 2025). They hypothesize that differences in sex and fetal growth are related to the development and functions of the placenta, steroid hormones, and genetic differences. The literature indicates that the interplay among hormones, chromosomes, neuroimmunity, and epigenetics actively contributes to differences between male and female brains. These differences manifest as variations in neurotransmitters, synapse formation, cell size in specific brain regions, and cell proliferation. Consequently, male and female brains exhibit distinct vulnerabilities to neurotoxic substances, including organophosphate pesticides (OPs) (Buralli et al. 2023). These studies corroborate previous findings in murine models of organophosphate pesticide exposure in pregnant rats, which showed decreased fetal growth and neurotoxicity (Yu et al. 2015). Furthermore, in non-mammalian embryonic models (amphibians and fish), there is clear evidence that developmental or growth retardation, through its effects on alkaline phosphatase in different tissues, negatively affects RNA/DNA synthesis. Regarding amphibians, studies report cellular and DNA damage, endocrine alterations, and growth impairment (Sabra and Mehana 2015; Brice et al. 2022).

Using biochemical parameters to assess nutritional status provides complementary information to that obtained through other assessment methods. Their interpretation is helpful at all stages of the process, as it allows the assessment of the state of different body compartments, guidance on the intake, absorption, or loss of specific nutrients, and calculation of nitrogen balance. However, it is essential to note that no biochemical determination is sufficient to assess nutritional status comprehensively (Moráis 2009; Kishor 2024). Biochemical analysis revealed that all children had an optimal nutritional status, with no significant differences between groups, except for ALP and GGT levels. Albumin, total proteín, calcium, and hemoglobin are markers of malnutrition when their levels are low(Szewczyk-Golec; however, the results of this study indicate that children indirectly exposed do not present biochemical alterations associated with malnutrition. Although urea and albumin values in indirectly exposed children were within the normal range, a significant increase was observed compared to control children (*p* < 0.001). A study conducted in the Gaza Strip (Yassin and Al-Shanti 2016) evaluated the impact of pesticide exposure on a group of rural workers, analyzing serum proteins and kidney function. The results showed that both albumin and urea were significantly increased in the group of rural workers compared to the control group.

On the other hand, the liver plays a fundamental role in maintaining homeostasis and is the main organ involved in the metabolism of xenobiotics, including pesticides (Dalmolin et al. 2020; Szewczyk-golec et al. 2025). For this reason, it was crucial to evaluate liver function in populations indirectly exposed to these compounds. Conventional liver function tests are widely used to assess hepatocellular and biliary system dysfunction by measuring serum levels of aminotransferases (ALT, AST) and cholestasis enzymes (γ-glutamyl transferase –GGT–), respectively. Liver enzyme analysis showed increased AST and GGT activity in 6% of exposed children. Several studies have shown that liver enzymes can be altered in agricultural worker populations (Lozano-Paniagua et al. 2021; Saghir et al. 2022; Dahlan et al. 2023; Ortiz-Delgado et al. 2025). These findings suggest a mild impairment of liver function and indicate that indirect exposure to pesticides could generate biochemical effects that, in the long term, could be associated with clinically significant hepatotoxicity. However, a review by Pizzolatto Dalmolin et al. (2020) points out that these enzymes do not show significant differences as biomarkers of the effect of organophosphate and carbamate pesticides (Dalmolin et al. 2020). Despite this, alterations in ALT and GGT were observed in the group of indirectly exposed children in this study, suggesting that indirect exposure to these pesticides could impact liver function. This highlights the importance of continuing to investigate the role of liver enzymes as potential biomarkers for monitoring environmental exposure to organophosphate and carbamate pesticides.

Although ALP values are elevated in unexposed children, this does not necessarily imply the presence of hepatobiliary disease. During the stages of most significant growth in childhood and adolescence, it is normal for these values to be up to five times higher than the reference range due to osteoblastic activity of the bone (Viñallonga and Bonjoch 2011; Bahnemiri et al. 2022). It has been reported that between 77 and 89% of the total serum ALP concentration in children is derived from bone tissue, and its levels can be influenced by factors such as age, sex, growth rate, and hormonal status (Turan et al. 2011). Since children undergo a process of bone growth, an increase in bone-derived ALP in serum is expected (Sánchez Rodríguez, et al. 2002; Bahnemiri et al. 2022). However, ALP values are significantly reduced (*p* < 0.001) in indirectly exposed children compared to unexposed children; this could be related to the decreased enzymatic activity of ALP due to indirect exposure to OPs, since these chemicals are associated with dysregulation of hormone metabolism-enzyme (Predieri et al. 2022) associated with alterations in skeletal formation. They act as bone toxins, affecting bone mass during adolescence by inhibiting bone deposition (Shulhai et al. 2024).

Nutrition, the hypothalamus-pituitary-liver, and the thyroid axis are the central regulators of linear growth and development in infancy and childhood ^47,48^; for this reason, the hormonal profile of all children participating in this study was analyzed. Endocrine gland disruption and malfunction have also been linked to organophosphates such as parathion and malathion (Kashyap et al. 2024). OPs insecticides and carbamates are endocrine-disrupting chemicals (EDCs) that can affect the hypothalamus and pituitary gland by inhibiting cholinesterase activity, thereby altering endocrine function (Campos and Freire 2016). IGF-1 regulates the action of GH. It involves tissue growth, metabolism, and various diseases, including metabolic and growth disorders (Huang et al. 2024). Our findings suggest that indirect exposure significantly impacts GH and IGF-1 levels, with a 2.55- and 1.28-fold reduction compared to the control group. Despite this decrease, the average values of both hormones remain within the normal range, indicating that although indirect exposure affects the regulation of these hormones, it does not necessarily imply a state of clinical deficiency. Several studies have associated OPs pesticide exposure with stunting and have found associations between exposure to these compounds and changes in circulating levels of thyroid hormones and/or TSH (Rachmi et al. 2016; Kartini et al. 2019; Diawara et al. 2024). Some of these findings are consistent with experimental data suggesting that pesticide exposure may reduce T_4_ and/or T_3_ levels, often accompanied by an increase in TSH, both in environmental and occupational exposures (Campos and Freire 2016). Regarding thyroid hormones, a generalized increase in fT4 and tT3 is observed in our study, with increases of 1.90 and 5.24 times, respectively, without significant differences between genders. This pattern suggests a possible activation of the hypothalamic-pituitary-thyroid axis in response to indirect exposure; the elevation of these hormones, especially in indirectly exposed children, could be related to compensatory or adaptive mechanisms in response to an environmental stimulus (Wafaa Kadhim Jasim 2023). The fact that their average values are slightly outside the normal range in this group requires further evaluation, as it could indicate an alteration in thyroid homeostasis with possible long-term clinical implications. These results are consistent with the study by Channa et al. (2021), who compared thyroid hormone and TSH levels in children living in rural and urban áreas (Channa et al. 2021). This study reported an increase in T₄ and T₃ in children from rural areas, with significant differences between groups but not between genders. No significant differences were observed in TSH, which could indicate that central regulation of thyroid function remains relatively stable despite changes in fT4 and tT3. Likewise, in our study, the absence of significant differences between genders and in the group-gender interaction suggests that exposure affects boys and girls similarly, without an important influence of sex on the hormonal response.

The results obtained in this study reinforce the usefulness of AChE and BuChE activity as biomarkers of exposure to OPs (Dalmolin et al. 2020). The significant reduction in AChE activity in indirectly exposed children compared to the control group suggests an inhibitory effect of environmental exposure to these compounds. This inhibition is consistent with the mechanism of action of OPs, which block the activity of cholinesterases, affecting cholinergic neurotransmission and potentially generating adverse effects on health. Regarding plasma cholinesterase (BuChE), a more pronounced inhibition was evident in the exposed group, consistent with previous studies indicating that BuChE may be a more sensitive marker of chronic pesticide exposure than AChE (Lionetto et al. 2013). Furthermore, the significant difference in BuChE activity between genders suggests that physiological factors, such as hormonal or metabolic differences between boys and girls, could influence susceptibility to enzymatic inhibition. The significant difference in specific AChE and hemoglobin values suggests that exposure to OPs could affect other hematological parameters, which merits further evaluation in future studies.

Future studies should explore these links through longitudinal analyses and assess possible underlying physiological mechanisms. One limitation in our study was the determination of GH, since this analyte fluctuates throughout the day. However, IFG-1 was also analyzed since its concentrations are more stable and reflect chronic GH secretion. Another limitation was the monitoring of pesticide metabolites in the samples obtained. However, determining the enzymatic activities of cholinesterases provides evidence of the effect of exposure to OPs. Despite our study's limitations, the findings underline the relevance of environmental exposure to pesticides for children's health and the importance of monitoring cholinesterase activity as an early indicator of toxicity.

We must acknowledge that the cross-sectional nature of the data does not allow for establishing causality, and future studies should explore these links through longitudinal analyses and evaluate possible underlying physiological mechanisms. However, this cross-sectional study facilitated a preliminary exploration of associations between variables, accurately reflecting the population situation at a specific time. Another limitation was the monitoring of pesticide metabolites in the samples obtained. Nevertheless, the determination of cholinesterase enzyme activities provides evidence of the effect of OP exposure. Despite the limitations of our study, the findings underscore the relevance of environmental pesticide exposure to children's health and the importance of monitoring cholinesterase activity as an early indicator of toxicity.

## Conclusions

The findings of this study underscore the relevance of environmental exposure to pesticides for children's health and the importance of monitoring cholinesterase activity as an early indicator of toxicity. It was evident that children of rural workers are chronically and indirectly exposed to OPs through household contamination. Short stature, elevated plasma cholinesterase levels, and some biomarkers and hormones suggest the effect of this chronic exposure to OPs on children's growth. The children's nutritional status was not affected by analysis of biochemical parameters and food safety studies, so growth disorders in children of farmers could be primarily associated with chronic exposure to these agrochemical agents. Monitoring these effects on physiological mechanisms and considering environmental strategies to reduce exposure to these endocrine and enzyme-disrupting agents is essential.

## Data Availability

The datasets generated during the current study are available from the corresponding author on reasonable request.
